# A retrospective cohort study on the association between poor sleep quality in junior high school students and high hemoglobin A1c level in early adults with higher body mass index values

**DOI:** 10.1186/s12902-022-00951-6

**Published:** 2022-02-15

**Authors:** Tomoko Kasahara, Hiromasa Tsujiguchi, Yumie Takeshita, Akinori Hara, Keita Suzuki, Nobuhiko Narukawa, Koichiro Hayashi, Masateru Miyagi, Atsushi Asai, Yohei Yamada, Haruki Nakamura, Fumihiko Suzuki, Kim-Oanh Pham, Toshio Hamagishi, Masaharu Nakamura, Aki Shibata, Yukari Shimizu, Thao Thi Thu Nguyen, Sakae Miyagi, Yasuhiro Kambayashi, Takayuki Kannon, Atsushi Tajima, Hirohito Tsuboi, Tadashi Konoshita, Toshinari Takamura, Hiroyuki Nakamura

**Affiliations:** 1 Department of Hygiene and Public Health, Graduate School of Advanced Preventive, Medical Sciences, Kanazawa University, 13-1 Takara-machi, Kanazawa, Ishikawa 920-8640 Japan; 2 Department of Hygiene and Public Health, Graduate School of Medical Science, Kanazawa University, 13-1 Takara-machi, Kanazawa, Ishikawa 920-8640 Japan; 3 Kanazawa University Advanced Preventive Medical Sciences Research Center, Takara-Machi 13-1, Kanazawa, Ishikawa 920-8640 Japan; 4 Department of Endocrinology and Metabolism, Graduate School of Medical Sciences, Kanazawa University, 13-1 Takara-machi, Kanazawa, Ishikawa 920-8640 Japan; 5 Community Medicine Support Dentistry, Ohu University Hospital, Koriyama, Fukushima 963-8611 Japan; 6 Faculty of Health Sciences, Department of Nursing, Komatsu University, 14-1 Mukaimotoori-Machi, Komatsu, Ishikawa 923-0961 Japan; 7 Faculty of Public Health, Haiphong University of Medicine and Pharmacy, 180000 Ngo Quyen, Hai Phong, Vietnam; 8 Innovative Clinical Research Center, Kanazawa University, 13-1 Takara-machi, Kanazawa, Ishikawa 920-8641 Japan; 9 Department of Public Health, Faculty of Veterinary Medicine, Okayama University of Science, 1-3 Ikoinooka, Imabari, Ehime 794-8555 Japan; 10 Department of Bioinformatics and Genomics, Graduate School of Advanced Preventive, Medical Sciences, Kanazawa University, 13-1 Takara-machi, Kanazawa, Ishikawa 920-8640 Japan; 11 Institute of Medical, Pharmaceutical & Health Sciences, Kanazawa University, Kanazawa, 920-1192 Japan; 12 Department of Endocrinology and Metabolism, University of Fukui Hospital, 23-3, Matsuokashimoaizuki, Eiheiji, Fukui, 910-1193 Japan; 13 Third Department of Internal Medicine, University of Fukui Faculty of Medical Sciences, 23-3, Matsuokashimoaizuki, Eiheiji, Fukui, 910-1193 Japan

**Keywords:** BMI, Glycated hemoglobin, Adolescent behavior, Sleep quality, Longitudinal study

## Abstract

**Background:**

Few epidemiological studies have been performed to clarify the association between glucose metabolism disorders in early adults (20 years old) and physiological and environmental factors, including body mass index (BMI) in junior high school days. Therefore, we examined the association between hemoglobin A1c (HbA1c) level and body size (BMI) in early adulthood and lifestyles, including sleep habits and BMI in junior high school days in Shika town, a small town in Japan, by conducting a retrospective cohort study.

**Methods:**

We examined the HbA1c levels and body size (BMI) of 99 early adults who turned 20 years old between 2016 and 2020 and were residing in Shika town, Ishikawa Prefecture. We obtained the information on lifestyles and living environment factors, including BMI, from a questionnaire survey conducted among the subjects during their junior high school days (13–15 years old) from 2009 to 2013.

**Results:**

No correlations were observed between the HbA1c levels and the BMI values of the early adults. A two-way analysis of covariance (with the HbA1c levels and BMI values of the early adults as main factors) of the body size and lifestyle habits of the junior high school students revealed that “sleep quality in junior high school” was significantly poorer in the high HbA1c group than in the low HbA1c group in the early adults with high BMI values only. This result was also supported by the logistic regression analysis result.

**Conclusions:**

The present results indicate that poor sleep quality in junior high school was associated with the high HbA1c levels of the early adults with higher BMI values, which suggests that good sleep quality in junior high school prevents the development of hyperglycemia. However, the present study did not find any relationship between early-adult BMI and HbA1c level.

**Supplementary Information:**

The online version contains supplementary material available at 10.1186/s12902-022-00951-6.

## Background

The prevalence of type 2 diabetes (T2DM) in adolescents and young adults is markedly increasing worldwide [1], particularly in the West Coast of the United States and Southeast Asia [2]. Environmental factors such as obesity, unhealthy diet, psychological stress, and physical inactivity, in addition to genetic factors, contribute to the development of T2DM [3–5]. Obesity has been identified as one of the contributing factors to T2DM. Although the rate of weight gain in Japanese people has plateaued, the prevalence of T2DM continues to increase [3, 6–8]. The mean body weight of the youth of Japan peaked in 1998–2006 and subsequently stabilized [7, 8]. Only a few longitudinal studies have demonstrated that lifestyle habits and body mass index (BMI) values of middle school students are risk factors of abnormal glucose metabolism [9, 10]. In 2016, Xi et al. [9] reported that low birth weight and central obesity in adolescents influenced the subsequent development of T2DM in young adults in China, which suggests that body weight control in the young effectively prevent and control T2DM in adulthood. Cross-sectional studies investigated sleep quality in the young as a risk factor of T2DM in adulthood [11, 12]. A relationship was found between sleep quality and insulin resistance that was dependent on adiposity [12]. However, no retrospective cohort study has yet been conducted to elucidate the relationship between sleep quality in the young and glucose metabolism in early adulthood. T2DM is partly caused by genetic factors and mainly by eating habits. Therefore, hypothesizing the effect of the living environment, BMI,and lifestyle factors such as sleep in junior high school students on their subsequent glucose metabolism, we conducted a retrospective cohort study using early adulthood hemoglobin A1c (HbA1c) level as the index.

## Methods

### Subjects

**Fig. 1:**
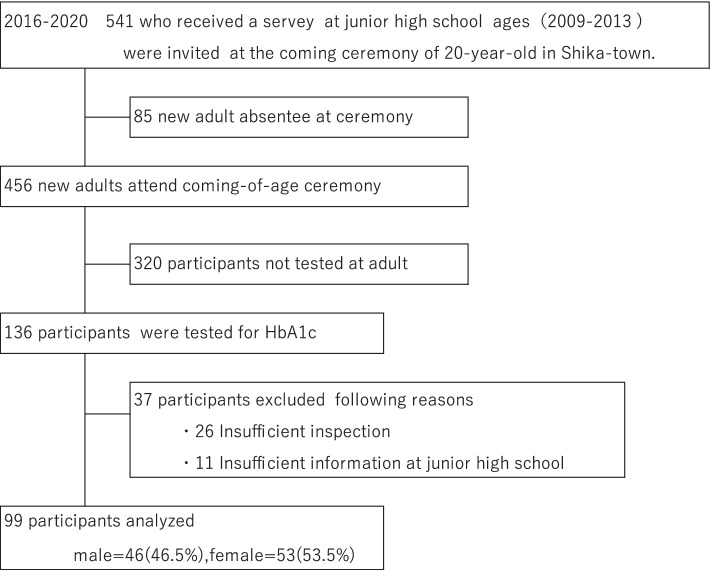
Participant recruitment chart

We conducted a retrospective study in Shika town, a rural area in the Noto Peninsula, Ishikawa Prefecture, Japan. Five hundred and forty-one people who participated in a self-reported questionnaire survey when they were enrolled in junior high schools in Shika between 2009 and 2013 were invited to the coming-of-age ceremony (2016–2020). A total of 405 people were excluded from the study if they did not attend the coming-of-age ceremony (85), did not receive a blood test for examining HbA1c, or agree to participate (320). Hence, 136 people participated in this study. However, during the analysis, 37 people were excluded (26 because of insufficient information, height, weight and HbA1c at the time of the examination and 11 of the questionnaire in junior high school). Figure 1 shows the inclusion criteria.

### BMI and lifestyle in junior high school

We collected data on body size assessed by school nurses, including junior high school height and weight, from school health records. BMI is calculated as weight (kg) divided by height squared (m^2^) [13]. We compared the BMI values and lifestyles of the analyzed and nonanalyzed subjects. Lifestyle habits were evaluated using a self-administered questionnaire based on the 2007 National Health and Nutrition Examination Survey by the Ministry of Health, Labour, and Welfare of Japan [11]. The questionnaire included items on dietary habits (snacking habit: less than once a week, every 2 or 3 days, or every day; dinner companion: none, siblings, or family), exercise habits (playing sports for one year or more: yes, no), sleeplessness, sleep duration (> 9, 8–9, 7–8, 6–7, 5–6, or < 5 h), sleep quality, and psychological stress (none at all, a little, some, or much). Sleep quality was evaluated using questions regarding whether the students had a sufficient amount of sleep to feel rested; the items were scored from 1 to 4 (1: sufficient, 2: to some extent, 3: poor, and 4: insufficient). Of these questions, the items on snacking habits, sleep duration, sleep quality, psychological stress, and family history of diabetes were adapted from the Ministry of Health, Labour, and Welfare’s National Health and Nutrition Survey [14, 15]. Furthermore, we added the question items on playing sports for one year or more, dinner companion, and the arranged items are snacking habits.

### Blood glucose levels and BMI in early adulthood

Early-adult HbA1c levels were measured using a quantitative immunoturbidimetric assay (A1C Gear System, Sakae Corporation, Gunma, Japan) using 1 μl of blood obtained from the fingertips of the subjects in early adult (20 years old). Information on height and weight was collected using a self-administered questionnaire. Body size was evaluated using early-adult BMI in the same manner as the junior high school age. The subjects were classified into two groups based on early-adult HbA1c levels as follows: a low early-adult HbA1c group (≤ 5.4%) and a high early-adult HbA1c group (> 5.4%). The cutoff point was defined according to the upper limit of the nondiabetic level in Japan [16–18]. The subjects were simultaneously categorized into two groups according to early-adult BMI as follows: a low early-adult BMI group (≤ 22 kg/m^2^) and high early-adult BMI group (> 22 kg/m^2^). Early-adult BMI of 22 kg/m^2^ is considered to have the lowest risk of lifestyle-related diseases by the Japanese Ministry of Health, Labour, and Welfare [19, 20].

### Statistical analysis

In the comparison of junior high school BMI/lifestyle between the 99 analyzed and 442 nonanalyzed subjects, the Student *t* test was used for continuous variables and the chi-square test was used for categorical variables.

A two-way analysis of covariance (ANCOVA) with adjustment for sex was used to examine the main effects of early-adult HbA1c level and BMI and their interaction with junior high school lifestyles. Multiple logistic regression analysis was performed to examine HbA1c levels after stratifying HbA1c levels, BMI values, and sleep qualities. Furthermore, a hierarchical regression model was applied to adjust for confounders. The Japanese version of the IBM SPSS Statistics Ver. 26 software for Windows (IBM, Armonk, NY, USA) was used for the statistical analyses. The significance level was set at 5%.

### Ethical consideration

This study was approved by the Ethics Committee of Kanazawa University (No. 1939, 1940). Written informed consent was obtained from all the subjects. The survey in junior high school was also conducted within the scope of the School Health and Safety Law in Japan.

## Results

### Subjects’ characteristics

Ninety-nine subjects consisting of 46 males (46.5%) and 53 females (53.5%) were included in the study. Table 1 shows the subjects’ characteristics and body sizes. The early-adult BMI (20 years old) of males were significantly higher than that of the female (*p* = 0.001). No differences in HbA1c level (*p* = 0.124) and diabetic family history (*p* = 0.445) were found.

**Table 1:** Subjects’ characteristics

	Total (*n* = 99)		Males (*n* = 46)		Females (*n* = 53)		*p* value
	Mean	SD	Mean	SD	Mean	SD	
Age	19.91	0.41	19.89	0.43	19.92	0.39	0.687
BMI in early adults (kg/m^2^)	21.96	3.87	23.43	4.95	20.68	1.88	**0.001**
HbA1c level (%)	5.24	0.30	5.29	0.28	5.19	0.32	0.124
Family history of diabetes, n (%)^a^	34(34.3)		14 (30.40)		20 (37.70)		0.445

The *p* values were from the Student *t* test for continuous variables and chi-square test for categorical variables. The *p* values < 0.05 are in bold. The continuous variables are presented as mean (SD). *Abbreviation*: *BMI* body mass index.^a^ 1: yes, 2: no

### Comparison of BMI and environmental factors between the 99 analyzed and 424 nonanalyzed junior high school students

Table 2 shows a comparison of BMI and lifestyle between the final analyzed (*n* = 99) and nonanalyzed junior high school students (*n* = 442). All the items, including BMI, were not significantly different between the analyzed and nonanalyzed students. The 99 subjects’ height and weight in junior high school and early adulthood were not significantly different from the Japanese average values [21, 22] (Table S-1).

**Table 2:** Comparison of BMI and environmental factors between the analyzed and nonanalyzed junior high school students

	Analyzed subjects		Non-analyzed subjects		*p* value
	all (*n* = 99)		all (*n* = 442)		
	Mean	SD	Mean	SD	
Sex (male), n (%)	46(46.5%)		211(47.7%)		0.819
Height (cm)	156.50	6.73	157.94	7.63	0.084
Weight (kg)	49.04	11.34	50.10	10.59	0.375
BMI (kg/m^2^)	19.87	3.42	19.98	3.41	0.764
Playing sports for > 1 year, n (%)^a^	85(85.90)		326(76.7%)		0.130
Snacking habits^b^	2.08	0.74	1.93	0.72	0.064
Dinner companion^c^	2.89	0.45	2.84	0.50	0.416
Sleep duration^d^	3.19	0.98	3.17	1.14	0.836
Sleep quality^e^	1.91	0.69	1.98	0.74	0.401
Psychological stress^f^	2.26	0.95	2.45	0.91	0.064

The *p* values were from the Student *t* test for continuous variables and chi-square test for categorical variables. The *p* values < 0.05 are in bold. Continuous variables are presented as mean (SD). *Abbreviation*: *BMI* body mass index

^a^1: yes, 2: no; ^b^1: less than once a week, 2: every 2 or 3 days, 3: every day; ^c^1: alone, 2: with siblings, 3: with family; ^d^1: > 9 h, 2: 8–9 h, 3: 7–8 h, 4: 6–7 h, 5: 5–6 h, 6: < 5 h; ^e^1: sufficient, 2: to some extent, 3: poor, 4: insufficient; ^f^1: none at all, 2: little, 3: some, 4: much

### Comparison of the BMI/environmental factors in junior high school between the early-adult HbA1c and BMI groups

Table 3 shows the results of the two-way ANCOVA with the early-adult HbA1c and BMI groups as fixed factors, sex as a covariate for body size/living environment, and junior high school lifestyle as a dependent variable. When the low early-adult HbA1c group was subdivided into two groups based on early-adult BMI, there were 50 participants in the low-BMI group and 27 in the high-BMI group. The subdivision of the high early-adult HbA1c group into two groups based on the early-adult BMI resulted in 15 participants in the low early-adult BMI group and 7 in the high-early-adult BMI group. The early-adult HbA1c group showed that “sleep quality” had a significant main effect (*p* < 0.001). In the high early-adult BMI group, “sleep quality” was significantly lower in the high than in the low early-adult HbA1c group (*p* = 0.001, the Bonferroni test; Figure S-1). Regarding junior high school lifestyle, a significant interaction was observed for “sleep quality” between the early-adult HbA1c and BMI groups (*p* = 0.024).

**Table 3:** Comparison of BMI and environmental factors in junior high school students between the early-adult HbA1c and BMI groups (ANCOVA)

	Total (*n* = 99)														
	HbA1c ≤ 5.4% (*n* = 77)						HbA1 c > 5.4% (*n* = 22)						*p* value		
	BMI ≤ 22 kg/m^2^ (*n* = 50)			BMI > 22 kg/m^2^ (*n* = 27)			BMI ≤ 22 kg/m^2^ (*n* = 15)			BMI > 22 kg/m^2^ (*n* = 7)					
	Mean	95% CI		Mean	95% CI		Mean	95% CI		Mean	95% CI		P1	P2	P3
		Lower	Upper		Lower	Upper		Lower	Upper		Lower	Upper			
Family history of diabetes	1.34	1.20	1.48	1.33	1.14	1.52	1.33	1.06	1.60	1.43	0.93	1.92	0.629	0.543	0.678
BMI in junior high school students (kg/m^2^)	18.44	18.04	18.83	22.88	21.05	24.70	18.19	17.44	18.95	22.11	19.56	24.67	0.479	** < 0.001**	0.715
Playing sports for > 1 year, n (%)^a^	1.18	1.07	1.29	1.19	1.03	1.34	1.00			1.00			0.059	0.823	0.982
Snacking habits^b^	2.03	1.76	2.31	2.00	1.66	2.34	2.10	1.69	2.51	1.83	1.40	2.26	0.885	0.553	0.561
Dinner companion^c^	2.86	2.72	3.00	2.85	2.64	3.00	3.00			3.00			0.249	0.885	0.975
Sleep duration^d^	3.18	2.89	3.47	3.19	2.86	3.52	3.33	2.75	3.91	3.00	1.93	4.07	0.803	0.988	0.515
Sleep quality^e^	1.90	1.70	2.10	1.70	1.46	1.94	2.00	1.70	2.30	2.57	1.84	3.30	** < 0.001**	0.108	**0.024**
Psychological stress^f^	2.24	1.96	2.52	2.44	2.13	2.76	2.07	1.49	2.64	2.14	1.15	3.13	0.533	0.214	0.810

Data were adjusted for sex. The *p* values < 0.05 are in bold. P1 represents the early-adult HbA1c group; P2, the early-adult BMI group; and P3, the interaction. Continuous variables are presented as mean (SD). *Abbreviation*: *BMI* body mass index, *CI* confidence interval, *HbA1c* hemoglobin A1c

^a^1: yes, 2: no; ^b^1: less than once a week, 2: every 2–3 days, 3: every day; ^c^1: alone, 2: with siblings, 3: with family; ^d^1: > 9 h, 2: 8–9 h, 3: 7–8 h, 4: 6–7 h, 5: 5–6 h, 6: < 5 h; ^e^1: sufficient, 2: to some extent, 3: poor, 4: insufficient; ^f^1: none, 2: little, 3: some, 4: much

### Logistic regression analysis of sleep in junior high school for early-adult HbA1c levels

After stratification according to early-adult BMI, we examined the effects of sleep in junior high school on early-adult HbA1c level by using a multiple logistic regression analysis, which included sex, “sleep quality,” exercise habits, snacking habits in junior high school, and family history of T2DM as independent variables (Table 4). Although junior high school sleep quality was not a significant variable in any model in the low early-adult BMI group, it significantly contributed to early-adult HbA1c level in any models in the high early-adult BMI group (OR: 10.928; 95% CI: 1.378–86.691; *p* = 0.024). These results imply that higher early-adult HbA1c level was only found in the high early-adult BMI group when junior high school sleep quality was poor, supporting the results of the two-way ANCOVA.

**Table 4:** A logistic regression analysis of early-adult hemoglobin A1c (HbA1c) levels

	BMI ≤ 22 kg/m^2^				BMI > 22 kg/m^2^			
	OR	95% CI		*p* value	OR	95% CI		*p* value
		Lower	Upper			Lower	Upper	
Model 1	1.506	0.599	3.783	0.384	7.297	1.297	41.048	**0.024**
Model 2	1.361	0.545	3.398	0.509	6.224	1.128	34.323	**0.036**
Model 3	1.362	0.544	3.412	0.510	10.560	1.335	83.525	**0.025**
Model 4	1.367	0.543	3.437	0.507	10.928	1.378	86.691	**0.024**

Model 1: sex and sleep quality in junior high school students; Model 2: sex, sleep quality, playing sports for one year or more in junior high school students; Model 3: sex, sleep quality, playing sports for one year or more, and snacking habits in junior high school students; Model 4: sex, sleep quality, playing sports for one year or more, snacking habits in junior high school students, and family history of diabetes. *BMI* body mass index, *OR* odds ratio, *CI* confidence interval

## Discussion

### Relationship between early-adult BMI and HbA1c level

A relationship between BMI and HbA1c levels in adulthood has been reported [20, 23–25]. However, this study did not find any relationship between early-adult BMI and HbA1c level. A Chinese twin cohort study with a 6-year follow-up showed that overweight/obesity at baseline was not a risk factor for developing prediabetes/diabetes [26], which is consistent with the findings of another study [27]. The Young Generation Group Health Examination conducted among adults in their 20 s and 30 s in Japan revealed that 64% of subjects in the high HbA1c group had a standard BMI [28], which is in accordance with the present results showing no relationship between HbA1c level and BMI in adulthood. Previous studies demonstrated that East Asians and Asian Americans, even those with low BMI values, were more likely to develop T2DM at a young age [29, 30]. The absence of a relationship between early-adult BMI and type 2 diabetes may be explained by the fact that the peak height growth in adolescence continues even at the age of 20 years, resulting in no increase in BMI [31].

### Relationship between junior high school sleep quality and early-adult HbA1c level

The present results show that junior high school quality of sleep was related to glucose metabolism in early adults. Early-adult HbA1c level was associated with “junior high school sleep quality” in the higher early-adult BMI group, regardless of sex, exercise habits, snacking habits, or family history of T2DM. Many cross-sectional studies have examined the relationship between sleep quality and early-adult HbA1c level [13, 30, 32–34]. This longitudinal study demonstrated a relationship between junior high school sleep quality and early-adult HbA1c level. According to a 12-year prospective cohort study in middle-aged individuals aged between 45 and 65 years in Sweden [35], the relative risk of T2DM was 2.8-fold higher for males with a short sleep time. Furthermore, reduced sleep time was associated with increased susceptibility to metabolic disorders such as obesity, T2DM, and high blood pressure [36]. Experimental studies [37, 38] also demonstrated that decreased leptin level, increased ghrelin level, increased appetite, decreased insulin sensitivity, and increased blood pressure were due to sleep deprivation. Intervention studies [39] designed to increase sleep volumes and improve sleep quality showed that these changes were useful as treatment and primary preventive measures for metabolic disorders [36].

The present results also indicated that poor sleep quality during adolescence resulted in higher early-adult HbA1c levels in overweight subjects only. A relationship between adolescent sleep quality and subsequent excessive weight gain has been reported [25, 40]. In this study, we found an interaction between early-adult overweight and junior high school sleep quality, with an odds ratio of 10.928 for developing hyperglycemia. This interaction may be explained by the relationship between sleep hormone melatonin level and nutrition. In addition to the correlation between melatonin secretion and the risk of developing T2DM [41], tryptophan, B vitamins, magnesium, zinc, folic acid, and polyunsaturated fatty acids are required for the synthesis of melatonin [41, 42]. Puberty is the most metabolically active period in the lives of young people, during which they require many nutrients such as proteins and minerals, including zinc, iron, magnesium, and vitamins [43]. In this study, the odds ratio for developing hyperglycemia because of sleep quality increased to 10.56 after excluding the effect of snacking habits. Therefore, there seems to be a relationship between snacking habits and sleep quality. This is consistent with a previous study conducted in eight cities in China, which demonstrated that a higher total energy intake associated with snacking habits leads to poor sleep quality in junior high school students [44, 45]. Malnutrition associated with an unbalanced diet during adolescence has been shown to be associated with excessive weight gain in subsequent life stages such as early adulthood [46–48]. Therefore, the lack of necessary nutrients, which is associated with poor sleep quality, may contribute to the development of hyperglycemia in early adults who are overweight.

### Limitations

This study has some limitations. The present results, which were obtained from a limited number of students in a small area of Japan, have a selection bias and thus cannot be generalized to other populations. The absence of a relationship between early-adult BMI and HbA1c level in our study might be due to our small sample size of 99 participants. Furthermore, junior high school sleep quality was self-reported in junior high school and was not based on objective indicators.

## Conclusion

The present results indicate that poor sleep quality among junior high school students was associated with high HbA1c levels in early adults with higher BMI values, which suggests that good sleep quality in junior high school prevents the development of hyperglycemia in early adulthood. However, in this study, we did not find any relationship between early-adult BMI and HbA1c level.

## Supplementary Information

**Additional file 1: Table S-1. **Height and weight of the subjects (comparison with the Japanese average values).

**Additional file 2: Figure S-1. **Interaction between the two hemoglobin A1c groups and two BMI groups of early adults.

## Data Availability

The data described in the manuscript will be made available upon request application and approval (Kanazawa University Ethics Committee; person in charge: Yuko Katsuragi, pub-jim2@staff.kanazawa-u.ac.jp).

## Abbreviations

ANCOVA: Two-way analysis of covariance
BMI: Body mass index
CI: Confidence interval,
HbA1c: Hemoglobin A1c
OR: Odds ratio
SD: Standard deviation
T2DM: Type 2 diabetes
