# Supplementary material for: A retrospective cohort study on the association between poor sleep quality in junior high school students and high hemoglobin A1c level in early adults with higher body mass index values
Source: BMC Endocr Disord. 2022 Feb 15;22:40. doi: 10.1186/s12902-022-00951-6 (PMC8845399; doi:10.1186/s12902-022-00951-6)
Supplement: Supplementary file 1 — Additional file 1: Table S-1. Height and weight of the subjects (comparison with the Japanese average values). [file 12902_2022_951_MOESM1_ESM.docx]

**Table S-1 Height and weight of the subjects (comparison with the Japanese average values)**

|  | | | Japanese average | | | Males (n = 46) | | | *p* value | Japanese average | | | Females (n = 53) | | | *p* value |
| --- | --- | --- | --- | --- | --- | --- | --- | --- | --- | --- | --- | --- | --- | --- | --- | --- |
|  |  |  | n | AV | *SD* | n | AV | *SD* |  | n | AV | *SD* | n | Mean | *SD* |  |
| Height (cm) | junior high school | |  |  |  | 46 | 157.67 | 8.60 | 0.162 |  |  |  | 53 | 155.48 | 4.37 | 0.842 |
|  |  | Grade 1 | 28 | 159.43 | 6.35 | 21 | 155.37 | 6.63 | 0.559 | 28 | 153.39 | 6.16 | 22 | 154.46 | 3.59 | 0.775 |
|  |  | Grade 2 | 24 | 164.49 | 5.97 | 20 | 158.30 | 9.29 | 0.522 | 22 | 154.10 | 6.14 | 27 | 156.59 | 4.92 | 0.623 |
|  |  | Grade 3 | 31 | 170.72 | 4.99 | 5 | 164.86 | 10.37 | 0.634 | 27 | 157.36 | 5.20 | 4 | 153.53 | 2.65 | 0.282 |
|  | early adult  (20 years old) | | 12 | 170.20 | 6.80 | 46 | 170.51 | 5.22 | 0.686 | 14 | 158.60 | 4.20 | 53 | 158.39 | 4.22 | 0.715 |
| Weight (kg) | junior high school | |  |  |  | 46 | 51.95 | 15.06 | 0.882 |  |  |  | 53 | 46.51 | 5.66 | 0.949 |
|  |  | Grade 1 | 28 | 48.24 | 6.58 | 21 | 46.94 | 9.31 | 0.896 | 28 | 44.50 | 6.49 | 22 | 44.38 | 4.94 | 0.981 |
|  |  | Grade 2 | 24 | 52.81 | 11.69 | 20 | 52.42 | 13.63 | 0.978 | 22 | 44.30 | 6.11 | 27 | 48.26 | 6.07 | 0.528 |
|  |  | Grade 3 | 31 | 58.79 | 8.90 | 5 | 71.16 | 25.34 | 0.679 | 27 | 50.13 | 6.97 | 4 | 46.48 | 2.29 | 0.251 |
|  | early adult  (20 years old) | | 12 | 57.00 | 8.80 | 46 | 68.06 | 14.12 | 0.186 | 14 | 49.00 | 5.30 | 53 | 51.88 | 5.06 | 0.332 |

The Japanese average body size was extracted from the following materials in the Ministry of Health, Labor and Welfare National Health and Nutrition Examination Survey Part 2 Results of Physical Condition Survey. The body size of the junior high school students in 2011 (Heisei 23) is shown (Table 11). The body size of the early adults in 2019 (Reiwa 1st year) is shown (Table 14). The p values < 0.05 are in bold. Abbreviation: SD, standard deviation
