# Supplementary material for: A retrospective cohort study on the association between poor sleep quality in junior high school students and high hemoglobin A1c level in early adults with higher body mass index values
Source: BMC Endocr Disord. 2022 Feb 15;22:40. doi: 10.1186/s12902-022-00951-6 (PMC8845399; doi:10.1186/s12902-022-00951-6)
Supplement: Supplementary file 2 — Additional file 2: Figure S-1. Interaction between the two hemoglobin A1c groups and two BMI groups of early adults. [file 12902_2022_951_MOESM2_ESM.docx]

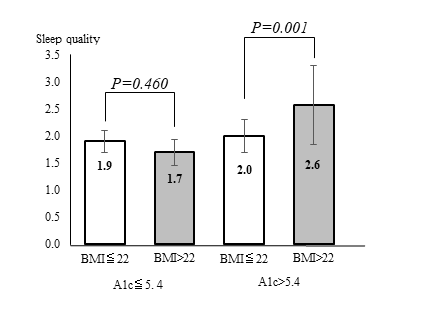


**Figure S-1 Interaction between the two hemoglobin A1c groups and two BMI groups of early adults.**

*Post hoc Bonferroni analysis. Adjusted for sex = 1.54, sleep quality, 1: enough rest, 2: good rest, 3: not much rest, and 4: no rest at all. Error bar: 95% confidence interval. Abbreviation: BMI, body mass index.
